# Expression of the Neuroblastoma-Associated ALK-F1174L Activating Mutation During Embryogenesis Impairs the Differentiation of Neural Crest Progenitors in Sympathetic Ganglia

**DOI:** 10.3389/fonc.2019.00275

**Published:** 2019-04-16

**Authors:** Lucie Vivancos Stalin, Marco Gualandi, Johannes Hubertus Schulte, Raffaele Renella, Olga Shakhova, Annick Mühlethaler-Mottet

**Affiliations:** ^1^Pediatric Hematology-Oncology Research Laboratory, DFME, University Hospital of Lausanne, CHUV-UNIL, Lausanne, Switzerland; ^2^Translational Oncology, Department of Hematology and Oncology, University Hospital Zürich, Zurich, Switzerland; ^3^Department of Pediatric Hematology, Oncology and SCT, Charité—Universitätsmedizin Berlin, Corporate Member of Freie Universität Berlin, Humboldt-Universität zu Berlin, and Berlin Institute of Health, Berlin, Germany; ^4^Berlin Institute of Health Berlin, Germany; ^5^German Cancer Consortium, Partner Site Berlin and German Cancer Research Center, Heidelberg, Germany

**Keywords:** ALK, neuroblastoma, differentiation, sympathetic ganglia, neural crest cells, SOX10, PHOX2B, mouse embryos

## Abstract

Neuroblastoma (NB) is an embryonal malignancy derived from the abnormal differentiation of the sympathetic nervous system. The Anaplastic Lymphoma Kinase (*ALK*) gene is frequently altered in NB, through copy number alterations and activating mutations, and represents a predisposition in NB-genesis when mutated. Our previously published data suggested that ALK activating mutations may impair the differentiation potential of neural crest (NC) progenitor cells. Here, we demonstrated that the expression of the endogenous *ALK* gene starts at E10.5 in the developing sympathetic ganglia (SG). To decipher the impact of deregulated ALK signaling during embryogenesis on the formation and differentiation of sympathetic neuroblasts, *Sox10-Cre;LSL-ALK-F1174L* embryos were produced to restrict the expression of the human ALK-F1174L transgene to migrating NC cells (NCCs). First, ALK-F1174L mediated an embryonic lethality at mid-gestation and an enlargement of SG with a disorganized architecture in *Sox10-Cre;LSL-ALK-F1174L* embryos at E10.5 and E11.5. Second, early sympathetic differentiation was severely impaired in *Sox10-Cre;LSL-ALK-F1174L* embryos. Indeed, their SG displayed a marked increase in the proportion of NCCs and a decrease of sympathetic neuroblasts at both embryonic stages. Third, neuronal and noradrenergic differentiations were blocked in *Sox10-Cre;LSL-ALK-F1174L* SG, as a reduced proportion of Phox2b^+^ sympathoblasts expressed βIII-tubulin and almost none were Tyrosine Hydroxylase (TH) positive. Finally, at E10.5, ALK-F1174L mediated an important increase in the proliferation of Phox2b^+^ progenitors, affecting the transient cell cycle exit observed in normal SG at this embryonic stage. Altogether, we report for the first time that the expression of the human ALK-F1174L mutation in NCCs during embryonic development profoundly disturbs early sympathetic progenitor differentiation, in addition to increasing their proliferation, both mechanisms being potential crucial events in NB oncogenesis.

## Introduction

Neuroblastoma (NB) is a pediatric malignancy of the sympathetic nervous system (SNS) which may arise in the adrenal medulla (47%) or along the entire sympathetic chain (53%) (1, 2). NB is considered to originate from a subset of neural crest cells (NCCs) committed to the sympathoadrenal (SA) lineage (3). However, recent studies described Schwann cell precursors (SCP) as a new cellular origin for the adrenal medulla, highlighting an additional cellular origin for adrenal NB (4).

NCCs are a transient and multipotent cell population of the developing embryos. During embryogenesis NCCs undergo epithelial-mesenchymal transition, and migrates toward their final destination where they differentiate into various cell types (5). The transcription factor (TF) Sox10 is expressed from ~E9 in trunk migrating NCCs where it mediates their survival, aids in the maintenance of multipotency and inhibits neuronal differentiation (6). NCCs of the sympathetic lineage follow a ventrolateral path to reach the vicinity of the dorsal aorta where bone morphogenetic proteins (BMPs) induce sympathetic neuron differentiation (5). This involves the expression of various TFs, including Paired-like homeobox 2a/b (Phox2a/b), Achaete-scute family BHLH transcription factor 1 (Ascl1), Insulinoma-associated 1 (Insm1), heart and neural crest derivatives expressed protein 2 (Hand2), and gata binding protein 3 (Gata3), acting as a network controlling the differentiation and specification of the NCCs into noradrenergic neurons (7). Early steps of noradrenergic differentiation are characterized by the upregulation of the enzymes tyrosine hydroxylase (TH) and dopamine β-hydroxylase (DBH), both involved in catecholamine biosynthesis, as well as other neuronal markers, including βIII-tubulin. The absence of even a single member of the TF network can lead to deregulation in sympathetic neuroblast proliferation, survival and/or noradrenergic differentiation (7).

NB is considered to constitute an embryonal tumor according to its tissue of origin, its early onset in childhood, and its ability to spontaneously regress. NB tumorigenesis is thought to be initiated *in utero* as an embryonal precancerous condition, and to arise through proliferation of sympathetic neuroblasts having escaped to apoptotic signals and to noradrenergic specification (8). For now, the specific mechanisms initiating NB are still largely unknown. Mutations or deregulations of key regulators of the sympathoadrenergic (SA) lineage may represent potential initial events for NB development, and MYCN, PHOX2B and ALK have previously been identified as potential first hits (8, 9).

The Anaplastic Lymphoma Kinase *(ALK)* gene is frequently altered in sporadic NB through amplification, copy number gain, overexpression, and mutations, and it has been defined as a predominant driver of familial NB (10, 11). The *ALK* gene encodes a receptor tyrosine kinase with an expression restricted to the central and peripheral nervous system (12–14). Point-mutations in its tyrosine kinase domain (TKD) have been reported in 8% of sporadic NB cases, with three major “hot-spots” mutations at position R1275, F1174, and F1245 (15–19). ALK-R1275Q, occurring in both familial and sporadic cases, and ALK-F1174L, restricted to sporadic cases, are reportedly the most potent activating mutations (19).

The expression of ALK-wt, ALK-F1174L, or ALK-R1275Q in NC progenitors or in sympathetic neuroblasts is not sufficient to drive NB tumor formation in absence of MYCN or other NB prototypical genetic alterations (20–23). However, ALK-F1174L and ALK-R1275Q have been described to strongly potentiate MYCN-mediated NB tumorigenesis in transgenic and knock-in (KI) animal models, highlighting their cooperation in sympathetic neuroblast progenitors and confirming their roles as possible initial events in NB initiation (20, 21, 23–25).

The precise role of ALK activating mutations during the initial stages of NB development remains unclear. However, the impact of ALK signaling in sympathetic neurogenesis has been investigated in several *in vitro* and *in vivo* models. ALK knockdown (shRNA) and its pharmacological inhibition (i.e., by TAE-684) were both associated with a decrease in the proliferation of chick sympathetic neuroblasts, while overexpression of ALK-wt or the ALK-F1174L or ALK-R1275Q variants increased their proliferation (26). In addition, ALK-F1174L induced a transient increase in the proliferation of chick primary sympathetic neuroblasts, followed by their differentiation after prolonged culture (27). Furthermore, an increased SG size was observed from embryonic to adult stages in ALK^−F1178L^ KI mice, associated with an enhanced proliferation of sympathetic neuroblasts and a prolonged duration of neurogenesis (23). However, in a recent study, we demonstrated that ALK-F1174L expression in the murine NC progenitor MONC-1 cell line generated undifferentiated tumors upon orthotopic implantation in nude mice. In contrast, MONC-1 parental cells gave rise to NB or osteochondrosarcoma, suggesting that ALK activating mutations may impair the differentiation capacity of NCCs (22).

In this study, we investigated the impact of the human ALK-F1174L mutation *in vivo* on sympathetic neuroblast differentiation using *Sox10-Cre;LSL-ALK-F1174L* mice. The aim was to improve our understanding of the implication of ALK deregulated signaling in the initial steps of NB genesis.

## Results

### ALK-F1174L Causes Embryonic Lethality When Expressed in NCCs Prior to SA Lineage Commitment

**Figure 1:**
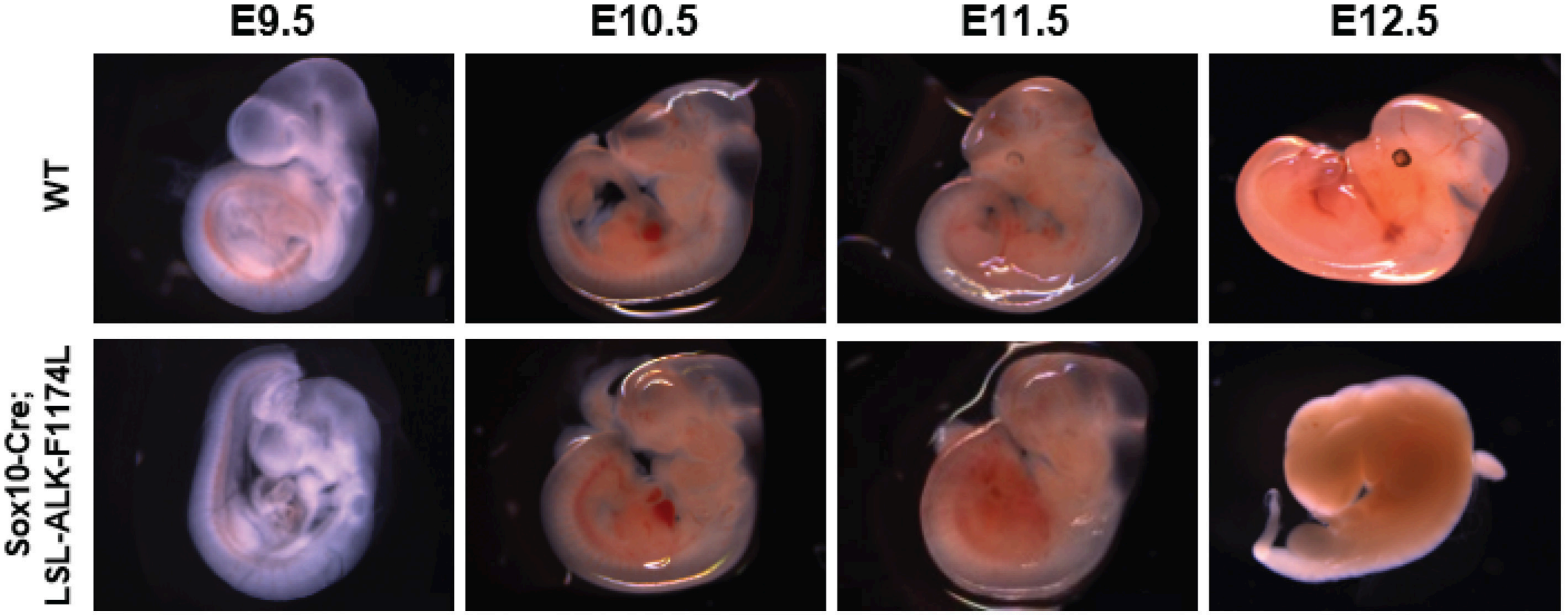
ALK-F1174L expression in Sox10^+^ cells NCCs mediates an embryonic lethality. Representative images of both *WT* and *Sox10-Cre;LSL-ALK-F1174L* embryo phenotypes for stages from E9.5 to E12.5. Embryos were genotyped by PCR as described in the Material and Method section.

To assess the impact of the human ALK-F1174L activating mutation on the development and differentiation of the SNS, *Sox10-Cre;LSL-ALK-F1174L* embryos were generated. This model allowed to restrict the expression of the ALK-F1174L variant to migrating Sox10^+^ cells before sympathetic lineage commitment. We observed that such lineage-specific expression of ALK-F1174L mediated an embryonic lethality in 100% of E12.5 *Sox10-Cre;LSL-ALK-F1174L* embryos (*n* = 4) (Figure 1). A similar phenotype was also observed in 1/8 and 1/3 *Sox10-Cre;LSL-ALK-F1174L* embryos at E10.5 and E11.5, respectively (data not shown).

### Endogenous ALK mRNA Is Detectable in Sympathetic Ganglia Starting at Stage E10.5

**Figure 2:**
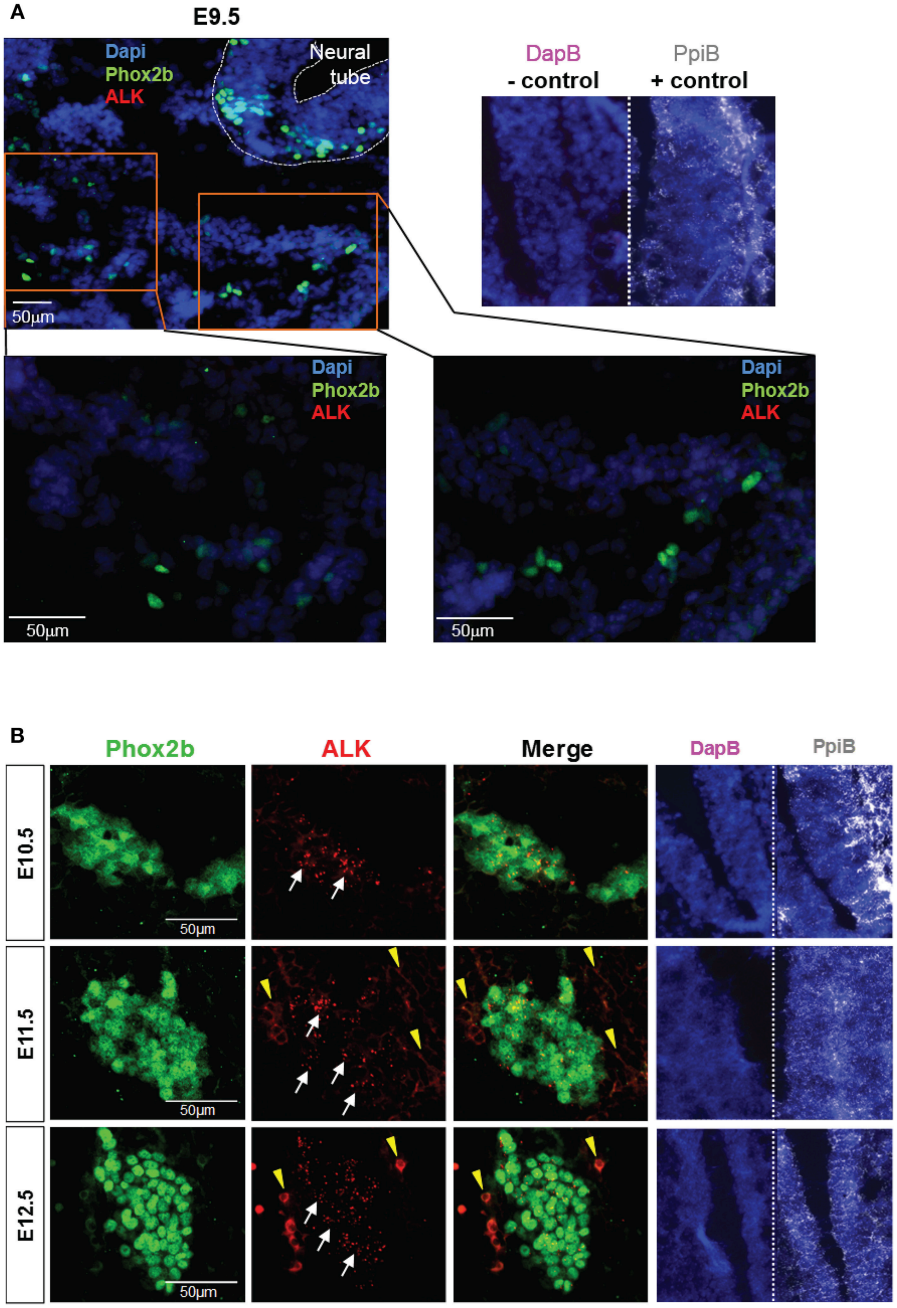
ALK mRNA is expressed from E10.5 in SG. Analysis of endogenous ALK mRNA expression in *WT* embryos. **(A)** Representative pictures of ALK ISH (red) staining of *WT* embryos at E9.5 (magnifications: x20 for top panel and x40 for lower panels). **(B)** Representative pictures of ALK ISH (red) staining in *WT* SG from E10.5 to E12.5 (×40 magnification). **(A,B)** Phox2b IF (green) staining was applied after ISH to highlight migrating sympathoblasts and SG. Specific ALK ISH signals (red, small dots) are identified by white arrows, and non-specific signals (diffuse cytoplasmic or membranous staining) by yellow arrowheads. Numbers of embryos analyzed: E9.5 *n* = 3, E10.5 *n* = 4, E11.5 *n* = 3, and E12.5 *n* = 2. Representative images of ISH with the positive control probe Mm-Ppib (white), showing mRNA integrity of the embryo sections, and the negative control DapB (pink), showing absence of the background due to molecule trapping in the tissue, are also displayed for each embryonic stages with Dapi staining (blue).

ALK is widely expressed in the developing nervous system. A previous report described the detection of ALK mRNA and protein in murine SG starting at E12.5 and E13.5 respectively (12). Here, we took advantage of a recently developed and highly sensitive *in situ* Hybridization (ISH) method, the RNAscope® assay (Advanced Cell Diagnostics, Inc), to precisely define the onset of ALK mRNA expression in the developing sympathetic chain of *WT* embryos. We observed ALK specific signals starting as early as E10.5 in the sympathetic trunk, while ALK mRNA was undetectable in migratory NCCs at E9.5 (Figure 2). Thus, in our *Sox10-Cre;LSL-ALK-F1174L* model the expression of the ALK-F1174L variant precedes the expression of the endogenous form by nearly 1.5 day.

### ALK-F1174L Affects Sympathetic Ganglia Formation and Early Sympathetic Progenitors Differentiation

Due to the early embryonic lethality, we focused our analysis of the impact of ALK-F1174L expression in NCCs on the development and differentiation of the sympathetic chain, as the initial structure of the adrenal medulla appears at E12.5. During sympathetic lineage specification around E9.5, migrating Sox10^+^/Phox2b^−^ NCCs transform into progenitors co-expressing Sox10 and Phox2b, and then the majority of SG cells switch to Sox10^−^/Phox2b^+^ immature sympathetic neuroblasts (7). While Sox10 expression is rapidly lost during neuronal differentiation, it is maintained in a minority of cells where it induces glial cell fate specification and differentiation (28, 29).

**Figure 3:**
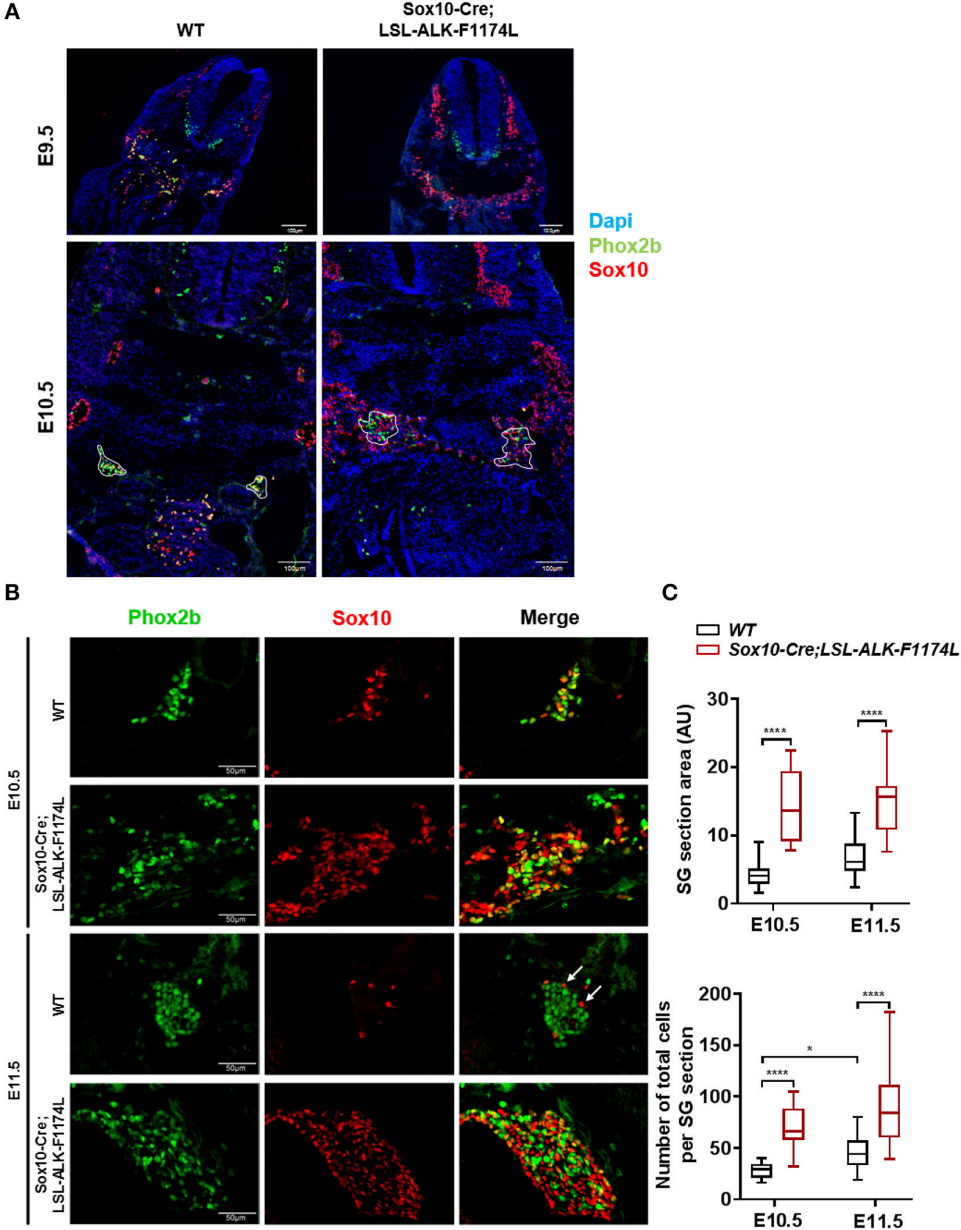
Increased SG size in *Sox10-Cre;LSL-ALK-F1174L* embryos. **(A)** Representative images of *WT* and *Sox10-Cre;LSL-ALK-F1174L* embryo sections stained for Sox10 (red) and Phox2b (green), with DAPI (blue) at E9.5 and E10.5 (x10 magnification). SG are surrounded by white circle. **(B)** Representative images of SG IF staining for Sox10 (red) and Phox2b (green) (x40 magnification). **(C)** Box-plot of SG section areas in arbitrary units (AU) (upper panel) and of the total numbers of cells per SG section (lower panel) at E10.5 and E11.5 in *WT* and *Sox10-Cre;LSL-ALK-F1174L* embryos (One-way Anova multiple comparison, ^****^p < 0.0001, ^*^p = 0.0306, not significant (ns) comparisons are not shown). Numbers of SG sections analyzed at E10.5 and E11.5, respectively: *WT n* = 22 and *n* = 23, *Sox10-Cre;LSL-ALK-F1174L n* = 14 and *n* = 16.

By IF co-staining for Sox10 and Phox2b, we first observed an elevated number of Sox10^+^ cells in *Sox10-Cre;LSL-ALK-F1174L* embryos at E9.5 and E10.5 (Figure 3A). Sox10^+^ NCCs still migrate ventro-laterally in *Sox10-Cre;LSL-ALK-F1174L* embryos. However, an abnormal number of cells were found in the dorsal root ganglia and in the region lateral to the dorsal aorta, where the ventral root and the sympathetic trunk formed indistinct structures. The expression pattern of the human ALK-F1174L transgene was similar to that of Sox10 in *Sox10-Cre;LSL-ALK-F1174L* embryos at E10.5 (Supplementary Figure 1). For further experiments, we focused our analyses at E10.5 and E11.5, as NCCs are still migrating toward the dorsal aorta at E9.5. We observed an alteration in the formation of the sympathetic chain in *Sox10-Cre;LSL-ALK-F1174L* embryos with an important increase in SG section areas at E10.5 and E11.5, by 3.3 and 2.2-fold, respectively, relatively to *WT* embryos (Figures 3B,C, upper panel). This observation was confirmed by an increased number of cells per SG section measured in *Sox10-Cre;LSL-ALK-F1174L* embryos relative to *WT* at both embryonic stages (Figure 3C, lower panel). Moreover, *Sox10-Cre;LSL-ALK-F1174L* SG displayed a pattern of disorganized architecture. Indeed, Sox10^+^ cells were found localized in the entire section of the ganglion at E11.5, while they were confined to the periphery in *WT* embryos (represented by arrows, Figure 3B), as previously described (28, 30).

**Figure 4:**
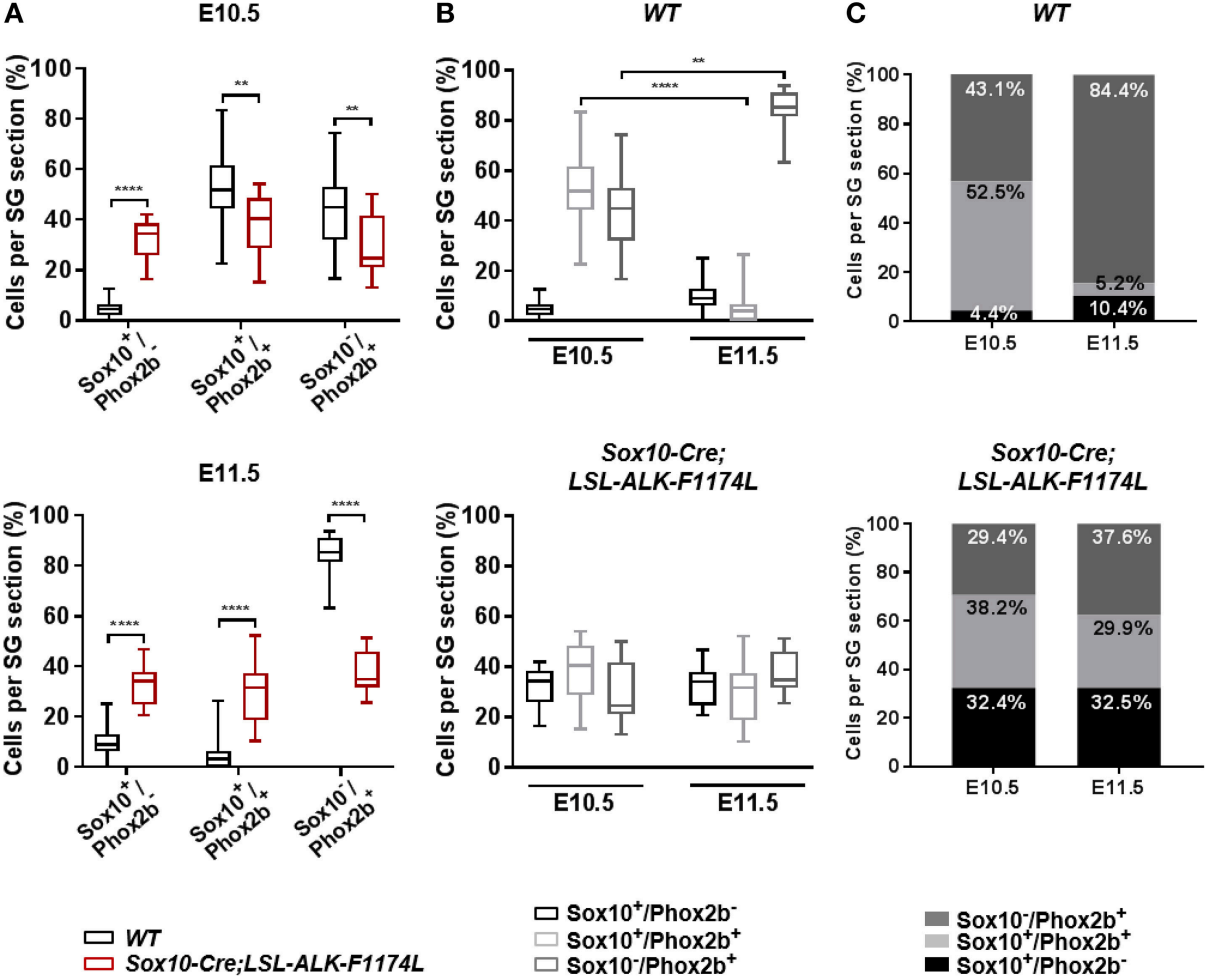
ALK-F1174L affects the differentiation of sympathetic progenitors. **(A)** The fractions of Sox10^+^/Phox2b^−^, Sox10^+^/Phox2b^+^, and Sox10^−^/Phox2b^+^ cell populations in *WT* and *Sox10-Cre;LSL-ALK-F1174L* embryos at E10.5 (top) and E11.5 (bottom) are shown in Box-plots (Mann Whitney test or unpaired *t*-test depending on data distributions, ^****^p < 0.0001, ^**^p < 0.01). **(B)** The evolution from E10.5 to E11.5 of the three cell populations in *WT* (top) and *Sox10-Cre;LSL-ALK-F1174L* (bottom) embryos are illustrated in Box-plots (Krukal-Wallis and One-way Anova respectively, paired comparisons, ^****^p < 0.0001, ^**^p = 0.0043, ns comparisons are not shown). Numbers of SG sections analyzed at E10.5 and E11.5, respectively: *WT n* = 22 and *n* = 23, *Sox10-Cre;LSL-ALK-F1174L n* = 14 and *n* = 16. **(C)** The mean percentages of each cell population are plotted in grouped representations at E10.5 and E11.5 for *WT* (top) and *Sox10-Cre;LSL-ALK-F1174L* (bottom) embryos.

Subsequently, we investigated the impact of the ALKF-1174L mutation on the transition from NCCs to sympathetic neuroblasts. We assessed the proportions of Sox10^+^/Phox2b^−^, Sox10^+^/Phox2b^+^, and Sox10^−^/Phox2b^+^ cells in SG sections co-stained for Sox10 and Phox2b. At E10.5, the percentage of Sox10^+^/Phox2b^−^ cells was markedly increased in *Sox10-Cre;LSL-ALK-F1174L* relative to *WT* SG (32.4 vs. 4.4%), while the proportions of Sox10^+^/Phox2b^+^ (38.2 vs. 52.5%) and Sox10^−^/Phox2b^+^ cells (29.4 vs. 43.1%) were significantly decreased (Figure 4A, upper panel). At E11.5, the fractions of Sox10^+^/Phox2b^−^ and Sox10^+^/ Phox2b^+^ cells were increased in *Sox10-Cre;LSL-ALK-F1174L* embryos relative to *WT* (32.5 vs. 10.4% and 29.9 vs. 5.2%, respectively), while the sympathetic neuroblast population was decreased (37.6 vs. 84.4%) (Figure 4A, lower panel). At E11.5, in *WT* embryos, as expected the majority of sympathetic progenitors (84.4%) had lost the expression of Sox10, while Sox10^+^/Phox2b^−^ and Sox10^+^/Phox2b^+^ cells represented 10.4 and 5.2%, respectively (Figures 4B,C, upper panels). In contrast, in *Sox10-Cre;LSL-ALK-F1174L* SG, no significant variations in the proportions of those three cell populations were observed between the two embryonic stages (Figures 4B,C, lower panels). Our results thus demonstrate that the ALK-F1174L mutation plays a decisive role in impairing the initial sympathetic differentiation by affecting the transition from NCCs to sympathetic neuroblasts.

### ALK-F1174L Impairs Noradrenergic Differentiation of Sympathetic Progenitors

**Figure 5:**
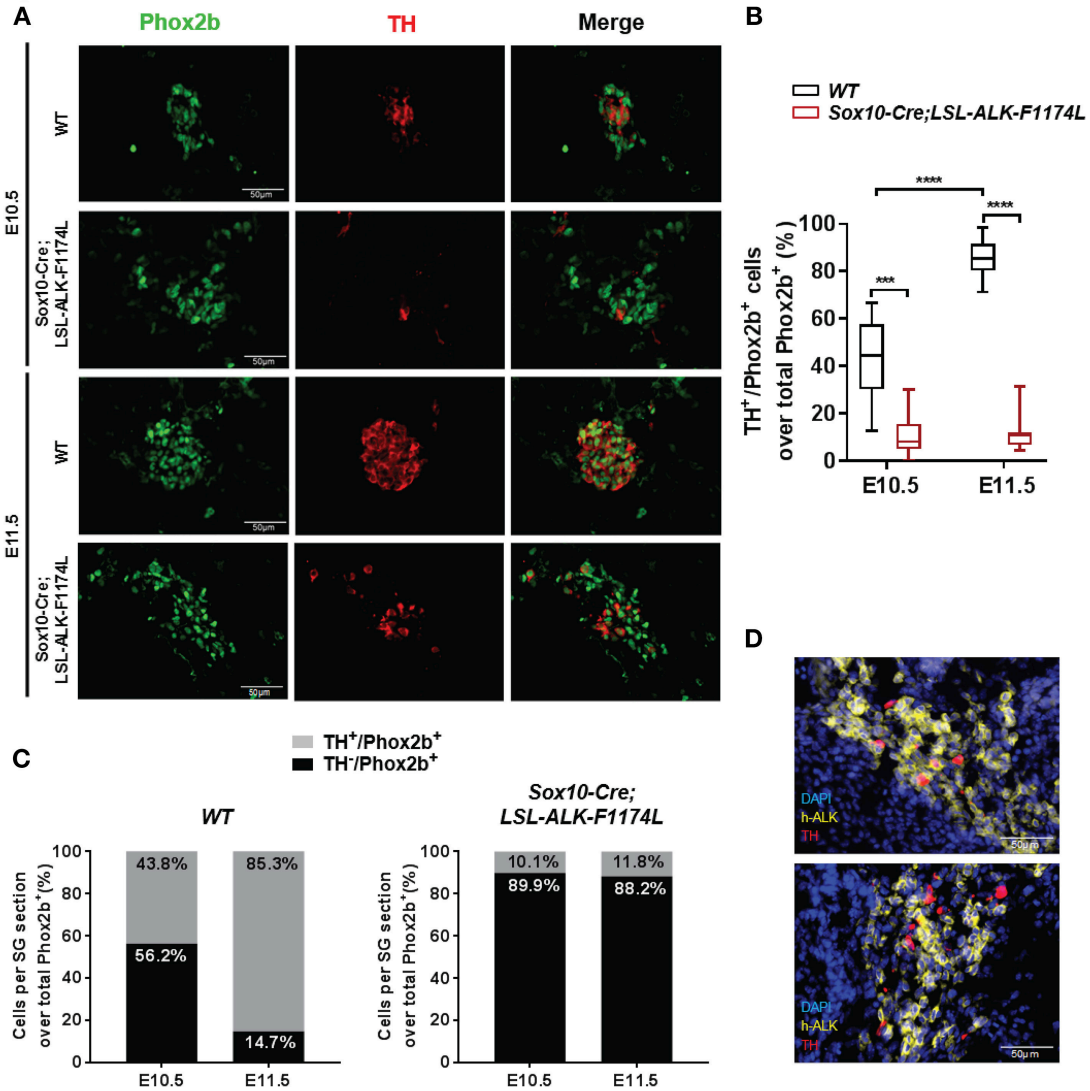
ALK-F1174L expression in sympathoblasts impairs noradrenergic differentiation. **(A)** Representative IF images of TH (red) and Phox2b (green) staining in *WT* and *Sox10-Cre;LSL-ALK-F1174L* embryos at E10.5 and E11.5 (x40 magnification). **(B)** The quantifications of the proportions of Phox2b^+^/TH^+^ cells over total Phox2b^+^ cells in *WT* and *Sox10-Cre;LSL-ALK-F1174L* embryos are illustrated in Box-plot for E10.5 and E11.5 (Kruskal-Wallis test, multiple comparisons, ^****^p < 0.0001, ^***^p = 0.0003, ns comparisons are not shown). Numbers of SG sections analyzed at E10.5 and E11.5, respectively: *WT n* = 30 and *n* = 30; *Sox10-Cre;LSL-ALK-F1174L n* = 18 and *n* = 16. **(C)** Mean percentages of TH^−^/Phox2b^+^ and TH^+^/Phox2b^+^ cell populations are plotted in grouped representations at E10.5 and E11.5 for *WT* (left panel) and *Sox10-Cre/LSL-ALK-F1174L* SG (right panel) embryos. **(D)** Representative IF images of double staining for human ALK (h-ALK) (yellow) and TH (red) in two *Sox10-Cre/LSL-ALK-F1174L* embryos at E10.5. Numbers of SG sections analyzed: *n* = 10.

To further assess the impact of the ALK-F1174L mutation on the noradrenergic differentiation of sympathetic progenitors, the expression of the early noradrenergic marker tyrosine hydroxylase (TH) was analyzed by co-staining with Phox2b. We quantified the fraction of TH^+^ cells over the total Phox2b^+^ cells, and we observed that TH was expressed in only rare cells in *Sox10-Cre;LSL-ALK-F1174L* SG at both E10.5 and E11.5 (Figure 5A). At E10.5, we detected only 10.1% of TH^+^/Phox2b^+^ neuroblasts in *Sox10-Cre;LSL-ALK-F1174L* SG, when compared to *WT* SG (43.8%) (Figure 5B). At stage E11.5, the fraction of TH^+^ cells increased in *WT* SG relative to E10.5, reaching 85.3% of Phox2b^+^ cells (Figures 5B,C, left panel). In contrast, in *Sox10-Cre;LSL-ALK-F1174L* SG, the proportion of TH^+^/Phox2b^+^ cells remained weak (11.8%) and equivalent to E10.5 (Figures 5B,C, right panel).

We investigated whether the rare TH-positive cells observed in *Sox10-Cre;LSL-ALK-F1174L* SG may result from NCCs that failed to effectively express the ALK-F1174L transgene. Therefore, *ALK-F1174L* embryos (E10.5) were labeled by IF staining to detect the human ALK (h-ALK) protein (Figure 5D). Surprisingly, 76.5% of TH^+^ cells were negative for h-ALK in the analyzed sections. This demonstrates that TH expression resulted from the lack of Cre-loxP-mediated recombination in NCCs and that TH was also drastically repressed in ALK-F1174L expressing sympathetic neuroblasts. These results indicate that the ALK-F1174L mutation plays a crucial role in blocking sympathetic neuroblasts differentiation and inhibiting noradrenergic differentiation of sympathetic progenitors at early developmental stages.

Noradrenergic differentiation is controlled by a TF network, comprising at the top of the cascade Phox2b and Ascl1, and immediately downstream Insm1 (7). To further understand the mechanisms mediating the blockage of noradrenergic differentiation observed in *Sox10-Cre;LSL-ALK-F1174L* embryos, we evaluated Ascl1 and Insm1 expression in SG at E10.5 by ISH. Specific Ascl1 and Insm1 signals were mainly localized within Phox2b^+^ cells in *WT* and *Sox10-Cre;LSL-ALK-F1174L* SG (Supplementary Figure 2). However, we observed a reduction in the number of ISH signals for both Ascl1 and Insm1 when reported to the SG section area in *Sox10-Cre;LSL-ALK-F1174L* SG relative to *WT* (Supplementary Figure 2). The decreased expression of both TF in SG may result from the incomplete sympathetic differentiation observed in *Sox10-Cre;LSL-ALK-F1174L* SG characterized by the reduced proportion of Phox2b^+^ cells. However, it may not explain the lack of noradrenergic differentiation mediated by the ALK-F1174L mutation.

### ALK-F1174L Affects Neuronal Differentiation

**Figure 6:**
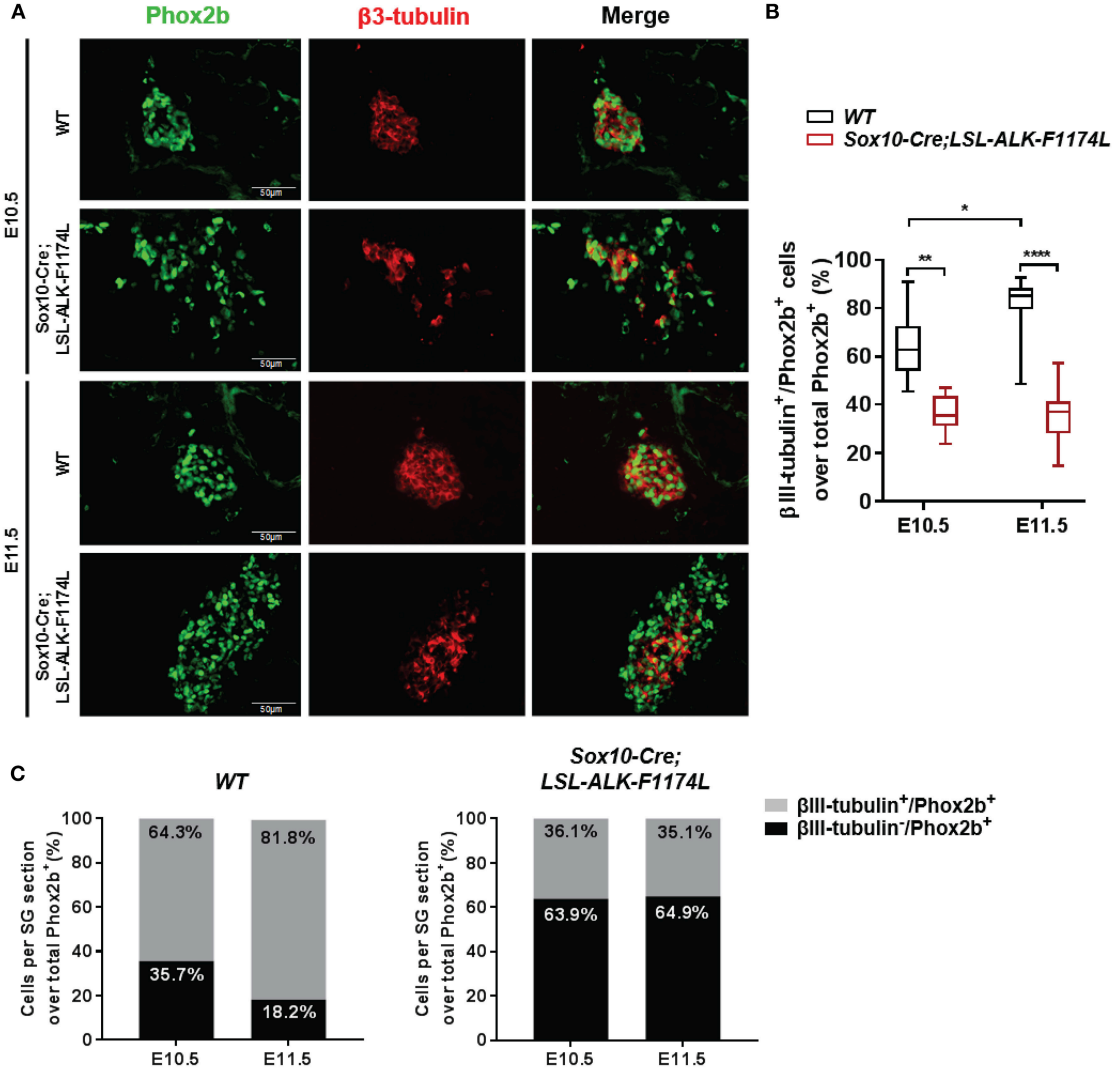
*Sox10-Cre;LSL-ALK-F1174L* SG displayed reduced neuronal differentiation. **(A)** Representative IF images of Phox2b (green) and βIII-tubulin (red) staining (x40 magnification). **(B)** Box-plot illustration of the fractions of βIII-tubulin^+^ cells over total Phox2b^+^ cells per section at E10.5 and E11.5 in *WT* and *Sox10-Cre;LSL-ALK-F1174L* embryos (Kruskal-Wallis test, paired comparisons, ^****^p < 0.0001, ^**^p = 0.0034, ^*^p = 0.023, ns comparisons are not shown). Numbers of SG sections analyzed at E10.5 and E11.5, respectively: *WT n* = 20 and *n* = 28, *Sox10-Cre;LSL-ALK-F1174L n* = 12 and *n* = 13. **(C)** The mean percentages of βIII-tubulin^−^/Phox2b^+^ and βIII-tubulin^+^/Phox2b^+^ cell populations are plotted in grouped representations at E10.5 and E11.5 for *WT* (left panel) and *Sox10-Cre;LSL-ALK-F1174L* (right panel) embryos.

We then assessed whether the ALK-F1174L mutation might also perturb the physiological acquisition of neuronal marker by double IF staining for Phox2b associated to the early pan-neural marker βIII-tubulin. The expression of the neuronal marker βIII-tubulin appears rapidly after Phox2b upregulation at E10.5 and was described both in Sox10^+^/Phox2b^+^ and Sox10^−^/Phox2b^+^ sympathetic progenitors (29). We quantified the fraction of βIII-tubulin^+^ cells over the total Phox2b^+^ cell population. The proportions of βIII-tubulin^+^ cells were reduced in *Sox10-Cre;LSL-ALK-F1174L* SG relative to *WT* SG at E10.5 (36.1 vs. 64.3%) and E11.5 (35.1 vs. 81.8%) (Figures 6A,B). Moreover, similarly to the acquisition of sympathetic and noradrenergic markers, no statistically significant changes were observed between E10.5 and E11.5 in *Sox10-Cre;LSL-ALK-F1174L* SG (36.1 and 35.1%, respectively) (Figures 6B,C, right panel), while the proportion of βIII-tubulin^+^ cells increased in *WT* SG (64.3–81.8%, respectively) (Figures 6B,C, left panel).

At E11.5, the proportion of the Phox2b^+^ cell population represented 89.6% in *WT* SG versus 67.5% in *Sox10-Cre;LSL-ALK-F1174L* SG (Figure 4C), corresponding to a 1.33-fold reduction. However, the proportion of βIII-tubulin^+^ cells at E11.5 dropped from 81.8 to 35.1% between *WT* and *Sox10-Cre;LSL-ALK-F1174L* SG, corresponding to a 2.3-fold reduction (Figure 6C). This suggests that the reduced expression of neuronal marker did not only result from the incomplete sympathetic differentiation, but also from a direct impact of ALK-F1174L on the regulation of neuronal differentiation. These data indicate that the ALK-F1174L mutation impairs the expression of the early neuronal marker βIII-tubulin, although its impact is minor than on TH expression.

### ALK-F1174L Increases the Proliferation of Undifferentiated Sympathetic Progenitors in Sympathetic Ganglia

**Figure 7:**
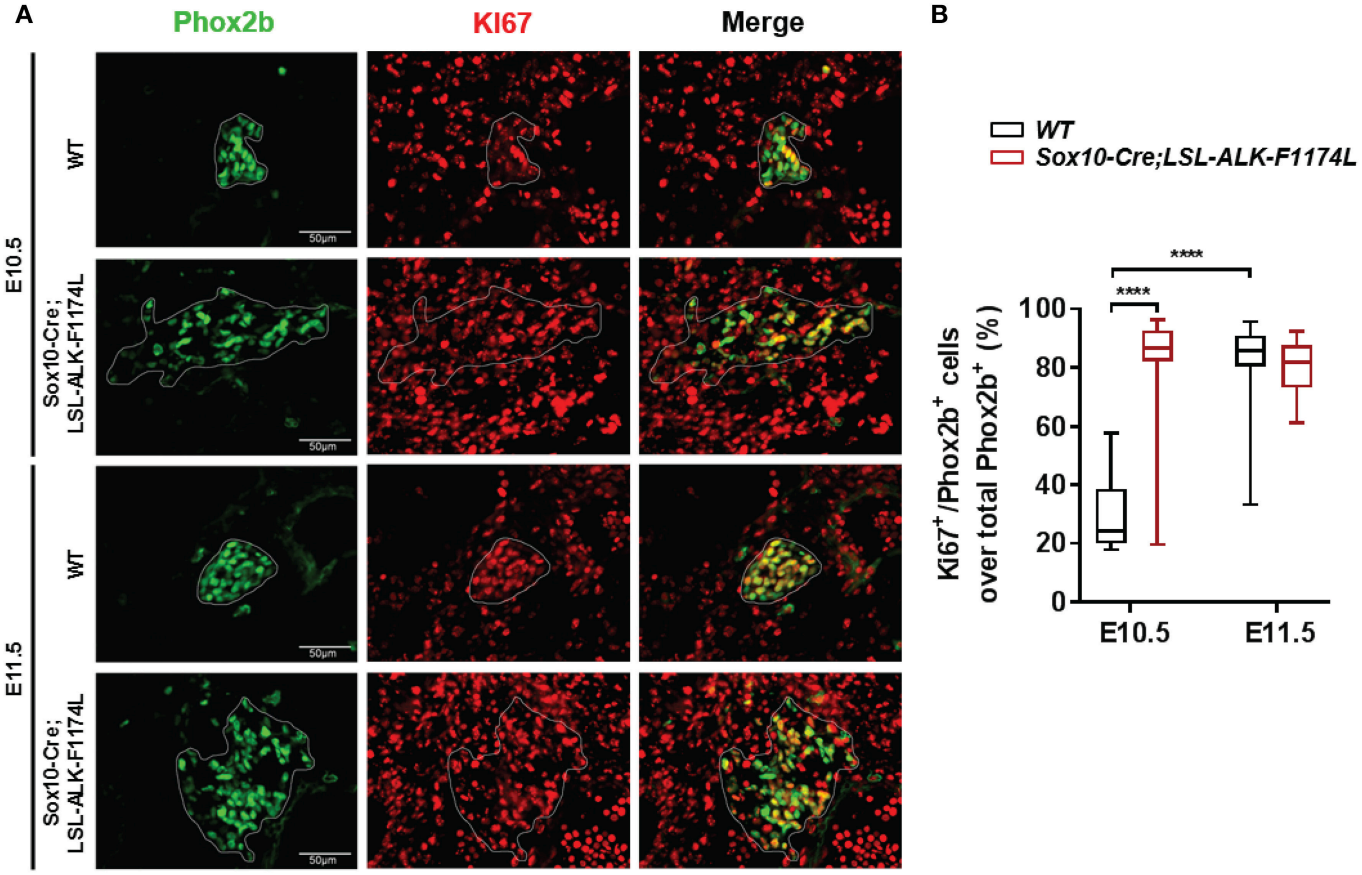
Increased proliferation of Phox2b^+^ cells in *Sox10-Cre;LSL-ALK-F1174L* SG. **(A)** Representative IF images of Phox2b (green) and Ki67 (red) staining at E10.5 and E11.5 in *WT* and *Sox10-Cre;LSL-ALK-F1174L* SG (x40 magnification). SG are surrounded by white circles. **(B)** Blox-plot representation of the fractions of Phox2b^+^/Ki67^+^ over total Phox2b^+^ cells in *WT* and *Sox10-Cre;LSL-ALK-F1174L* SG at E10.5 and E11.5 (Kruskal-Wallis test, multiple comparisons, ^****^p < 0.0001, ns comparisons are not shown). Numbers of SG sections analyzed at E10.5 and E11.5, respectively: *WT n* = 24 and *n* = 22, *Sox10-Cre;LSL-ALK-F1174L n* = 16 and *n* = 16.

The abnormally increased SG size observed in *Sox10-Cre;LSL-ALK-F1174L* embryos could be explained by an increased proliferation of NCCs and/or sympathetic progenitors. To verify this hypothesis, we analyzed the proportion of proliferating Phox2b^+^ cells in *WT* and *Sox10-Cre;LSL-ALK-F1174L* SG by Phox2b and Ki67 co-staining. Results showed that the proportion of Ki67^+^ cells over total Phox2b^+^ progenitors was significantly increased in *Sox10-Cre;LSL-ALK-F1174L* SG compared to *WT* SG (mean of 80 vs. 29.2%) at E10.5 (Figures 7A,B). At E11.5, the proportion of proliferating Phox2b^+^ cells, mainly composed of sympathetic neuroblasts, increased in *WT* SG (82.2%) reaching the proportions observed in *Sox10-Cre;LSL-ALK-F1174L* SG at both E10.5 and E11.5 (Figures 7A,B). These results indicate that ALK-F1174L expressing neuroblasts at E10.5 display an markedly high proliferation rate.

## Discussion

NB is thought to derive from aberrant NC development during embryogenesis. ALK mutations have been identified as predisposing to NB and may constitute a “first hit” in NB genesis. Here we studied the involvement of deregulated ALK signaling on sympathetic ganglia formation and neuroblast differentiation during early embryonic development.

In our *Sox10-Cre;LSL-ALK-F1174L* mouse model, as ALK-F1174L transgene expression was not restricted to the SA lineage-committed NCCs, the embryonic lethality may result from ALK-F1174L-mediated effects on Sox10^+^ migrating NCCs and their progeny. Alternatively, it could be caused by the absence of TH expression, as norepinephrine deficiency has been described to mediate embryonic death at mid-gestation (31–33).

ALK deregulated signaling in *Sox10-Cre;LSL-ALK-F1174L* embryos did not impair the migration of NCCs as Sox10^+^ cells were still able to migrate ventrolaterally. Nonetheless, the abnormal number of cells observed in the ventral roots and regions lateral to the dorsal aorta, at E9.5 and in particular at E10.5 strongly suggest that ALK-F1174L elicits an abnormal proliferation of the Sox10^+^ NCCs at early embryonic stages.

In *WT* embryos, the proportionality of the three cell populations were in line with previously published data, reflecting a physiological sympathetic differentiation (7, 29, 34). In contrast, ALK-F1174L caused a transitional block in NCCs as the sympathetic specification was only incompletely initiated, and a large proportion of Sox10^+^/Phox2b^−^ NCCs remained present both at E10.5 and E11.5. This observation may result from an ALK-F1174L-mediated inhibition of NCCs responsiveness to BMPs signaling. Alternatively, as an abnormal number of Sox10^+^ NCCs reached the vicinity of the dorsal aorta in *Sox10-Cre;LSL-ALK-F1174L* embryos, the amount of BMPs may not be sufficient to stimulate the integrality of these NCCs. Moreover, a complex reciprocal and dose-dependent inhibition loop between Sox10 and Phox2b is involved in sympathetic ganglia differentiation: at higher levels, Sox10 inhibits Phox2b expression, while at lower levels it is required for the initial upregulation of Phox2b (35). In addition, Phox2b negatively controls Sox10 expression (30, 36) but has also been shown to upregulate its own expression (37). In our model, the maintenance of Sox10 expression in *Sox10-Cre;LSL-ALK-F1174L* SG suggests that ALK-F1174L may affect the regulatory loop between Sox10 and Phox2b.

Furthermore, we observed a decrease in cells expressing βIII-tubulin, reflecting the incomplete acquisition of neuronal properties in *Sox10-Cre;LSL-ALK-F1174L* SG. This effect was not only due to the reduced proportion of sympathetic neuroblasts but also involved a direct ALK-F1174L-mediated inhibition of neuronal differentiation. Importantly, we highlight an important block in the acquisition of the noradrenergic marker TH in *Sox10-Cre;LSL-ALK-F1174L* SG. This indicates that even cells responding to BMPs signaling were unable to acquire noradrenergic features. Further experiments are necessary to elucidate the specific molecular mechanisms driving these observations, but one hypothesis would be that ALK-F1174L dysregulates the TF network essential for the initiation of noradrenergic differentiation. However, our data suggest that Ascl1 and Insm1 may not be involved as these TF were globally detected in Phox2b^+^ cells in *Sox10-Cre;LSL-ALK-F1174L* SG. Our finding that ALK blocks differentiation of NCCs during embryogenesis is in accordance with previous reports in tumors derived from the NC progenitor cell lines, MONC1 and JoMa1, expressing ALK mutations (22, 38). However, an opposite role for ALK in mediating neurite outgrowth was reported in rat pheochromocytoma (PC12) cells, the human SK-N-SH NB cell line and sympathetic neuroblasts (27, 39, 40). In addition, a more differentiated phenotype was observed in NB tumors expressing a constitutively activated ALK variant in combination with MYCN when compared to MYCN-only driven tumors (23). These contrasting findings suggest that sympathetic neuroblasts may respond differentially to ALK-mediated signaling depending on their maturational stage, as previously suggested (27, 41).

Embryonal tumors, such as NB, are thought to originate from an excessive proliferation of the tissue of origin prior to birth, coupled with an incomplete differentiation. In *Sox10-Cre;LSL-ALK-F1174L* embryos, the reduced sympathetic differentiation was associated with an increased expansion of Sox10^+^ NCCs, an enhanced proliferation of Phox2b^+^ progenitors at E10.5, and an enlarged size of *Sox10-Cre;LSL-ALK-F1174L* SG. These data are in accordance with the ALK^−F1178L^ KI model, which displayed enlarged stellate and superior cervical ganglia at birth and in adult mice, together with an elevated proliferation of neuroblasts at E14.5, and at birth (23). Similar involvement of ALK signaling in promoting proliferation of NCC progenitors and sympathetic neuroblasts has also previously been described in various models (22, 26, 27, 38).

Interestingly, the control of neurogenesis in the SNS is unique among neuronal cells as it is not linked to exit from cell cycle. Indeed, neuroblasts of the SNS continue to divide after initial neuronal differentiation and display two phases of proliferation (26, 42, 43). The first phase consists of an initial expansion of NC progenitors, which comprise both Sox10^+^/Phox2b^−^ and Sox10^+^/Phox2b^+^ cells. Then, a transient cell cycle exit occurs at E10.5 in cells displaying Sox10 downregulation together with upregulation of neuronal (βIII-tubulin, Hu) and noradrenergic (TH) markers (29, 43). Subsequently, the majority of immature noradrenergic neuroblasts (TH^+^) undergo a second wave of proliferation starting at E11.5 in mouse (7, 29). Our results in *WT* embryos are consistent with these reports, as 29% of Phox2b^+^ cells were proliferating at E10.5 and 82% at E11.5. In contrast, the abnormal and high proliferation index of Phox2b^+^ cells (80%) in *Sox10-Cre;LSL-ALK-F1174L* SG at E10.5, suggests that the ALK-F1174L mutation may prevent neuroblast progenitors from exiting the cell cycle. The mechanisms mediating the transient cell cycle exit are not clearly understood. However, it has been suggested that Sox10 might drive the proliferation of NCCs, and that the loss or the reduction of Sox10 expression could be involved (29, 35). In addition, only the cells with reduced or complete Sox10 downregulation expressed βIII-tubulin and TH at E10.5, and they displayed a proliferation arrest (29). We can thus hypothesize that in *Sox10-Cre;LSL-ALK-F1174L* SG, the persistently elevated expression of Sox10 may impede neuroblast progenitors from exiting the cell cycle and therefore prevent their differentiation.

It is worth noting that neuroblastoma-like premalignant lesions were identified in human fetuses or neonates via mass-screening and autopsies in prior independent studies (8, 44, 45). As they occur at much higher rates than the incidence of NB, it was speculated that the majority might indeed regress or mature spontaneously (8, 46, 47). Similarly, clusters of hyperplastic proliferating βIII-tubulin and TH negative cells were described in SG as well as in adrenal glands of newborn WT mice (34, 48). These hyperplastic clusters in SG were found to disappear postnatally at 14 days in WT mice, while they increased in size during the first postnatal weeks in the TH-MYCN NB mouse model, and even gave rise to NB-like tumors (48). Such precancerous clusters were shown to express Phox2b in the TH-MYCN mice, but lacked TH expression (49). These cells could represent the cellular origin of NB. Indeed, NB tumors, derived from TH-MYCN mice or from xenografts of human NB cell lines, were found mainly composed of proliferating undifferentiated Phox2b^+^/TH^−^ sympathetic progenitors (49). Importantly, these cells constitute large fractions of SG in our *Sox10-Cre;LSL-ALK-F1174L* model. We can thus hypothesize that deregulated ALK signaling, due to mutation or overexpression, in rare NCCs during embryonic development could promote the expansion of precursors of preneoplastic lesions. Further studies should focus on whether such precancerous states are also more prevalent and biologically persistent in the ALK^−F1178L^ or the AKL^−R1279Q^ KI model after birth.

The limitations of the experiments presented herein are mostly due to the fact that the expression of the ALK-F1174L variant in our model is anterior by nearly 1.5 days and possibly different i.e., more elevated than the one resulting from the endogenous gene. Thus, the observed ALK-F1174L-mediated impacts are more striking in our model as compared to the ALK^−F1178L^ KI model. Indeed, this KI model displayed lethality between 24 and 48 h after birth in homozygous mice (23). However, increased SG sizes and neuroblast proliferation were also observed in the ALK^−F1178L^ KI model (23). In addition, the early lethality of our model also limited the time-frame for the analyses relating to the differentiation of SG. We cannot exclude that the expression of the ALK-F1174L variant could mediate a delay in SA differentiation, as observed in murine Ascl1 and Insm1 knock-out models (50–52). However, as the transition from NCCs to sympathetic neuroblasts occurs between E9.5 and E11.5, we could precisely study the impact of ALK-F1174L during the initial phase of sympathetic lineage specification. Moreover, the ALK-F1174L mutation is not found in familial NB, but restricted to sporadic cases (19), suggesting that activation of ALK above a critical threshold might not be compatible with survival. Nevertheless, although no mutations at positions F1174 and F1245 were identified in familial NB cases, *de novo* germline mutations, ALK-F1174V and ALK-F1245V, were reported in two independent cases of congenital NB associated with CNS developmental defects (53). This potentially indicates that activating mutations at position F1174 can occur *de novo* in the germline and result in NB and severe CNS abnormalities. Finally, whilst our findings are mostly descriptive, this study opens new avenues for further mechanistic investigations to decipher the precise molecular underpinnings of ALK-mediated perturbations of the proliferative and differentiative checks and balances in place in SG, as well as in adrenal chromaffin cells. This is of particular interest, as the majority of adrenal chromaffin cells were recently described to originate from SCP rather than from a common SA progenitor (4).

**Figure 8:**
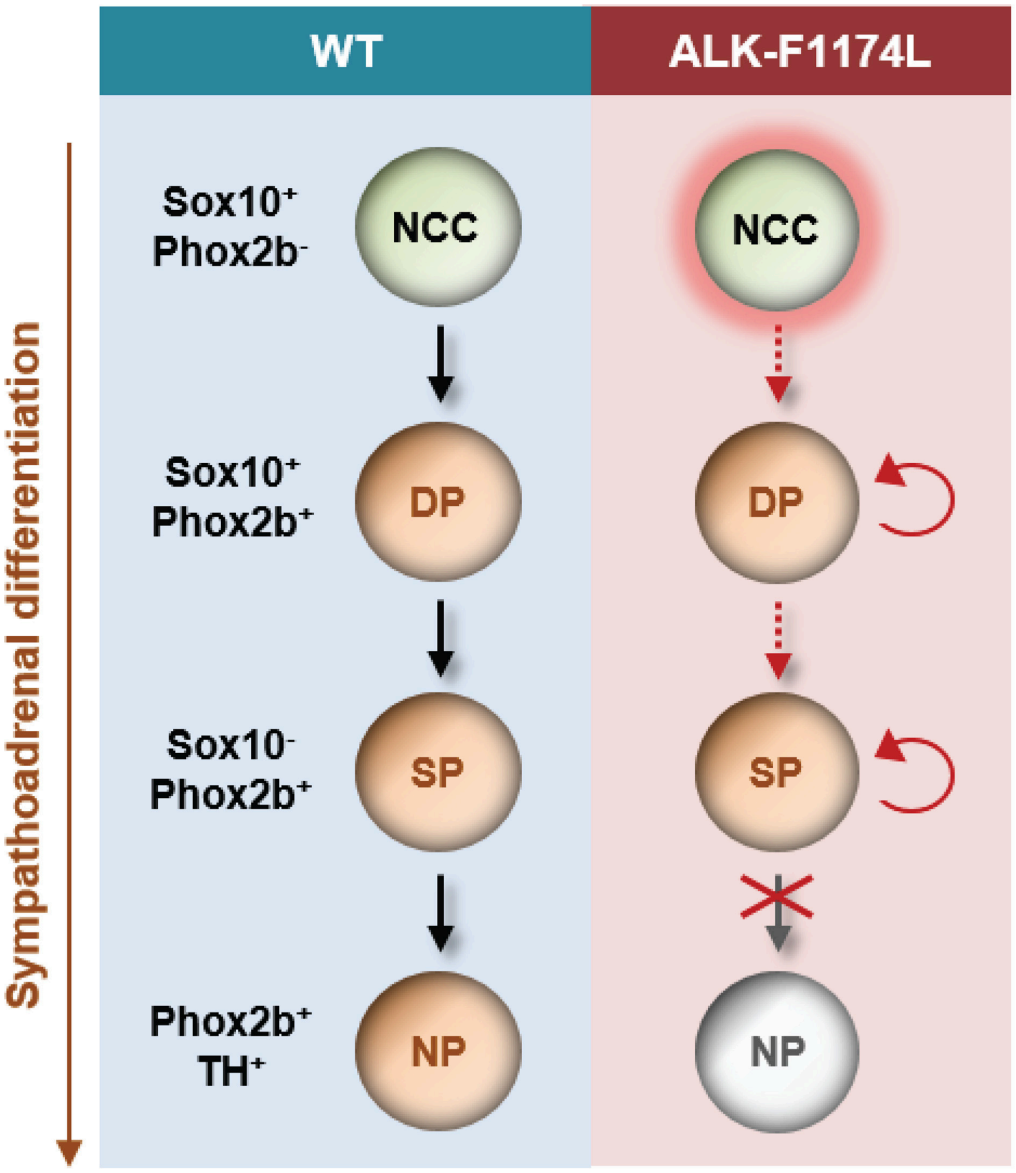
Schematic model proposed for the impact of the ALK-F1174L mutation on sympathetic differentiation. The expression of the ALK-F1174L mutation in NCCs affects various steps of the sympathetic differentiation during SA lineage development. ALK-F1174L increases the number of NCCs at early embryonic stages (marked by red halo). The transition from NCCs to Sox10^+^/Phox2b^+^ progenitors, and further to sympathetic progenitors is incomplete in *Sox10-Cre;LSL-ALK-F1174L* embryos (red dashed arrows). The expression of Sox10 is maintained in a large proportion of cells, while the cell population upregulating Phox2b is reduced, and no variation occur between embryonic stages. The Sox10^−^/Phox2b^+^ sympathetic progenitors do not acquire noradrenergic properties highlighting that ALK-F1174L hinders their maturation into noradrenergic neurons. The proportion of proliferating Phox2b^+^ cells is increased in *Sox10-Cre;LSL-ALK-F1174L* SG at E10.5 (red circular arrows). Thus, ALK deregulation leading to reduced SA differentiation and proliferative excess may be an essential path for NB initiation. NCC, neural crest cell; DP, differentiating progenitor; SP, sympathetic progenitor; NP, noradrenergic progenitor.

In conclusion, our study is the first demonstration of a role for the ALK-F1174L mutation in disturbing the balance between proliferation and differentiation in the sympathetic lineage *in vivo* at early embryonic stages. This occurs by (a) an increase in neuroblast proliferation, and (b) a block in neuronal and noradrenergic differentiation (Figure 8). Our data strongly suggest that dysregulated signaling of ALK during embryogenesis promotes a precancerous state, and thus may represent an initial and developmental path for NB oncogenesis.

## Materials and Methods

### Generation of LSL-ALK-F1174L Mice

The human ALK cDNA, harboring the ALK F1174L mutation (synthetized by GenScript), was cloned downstream of a chicken actin gene (CAG) promoter followed by loxP-flanked strong transcriptional termination site (LSL). The transgene was placed upstream of an internal ribosome entry site (IRES) and a second open reading frame coding for the luciferase gene (Fluc) in a proprietary plasmid (Taconic-Artemis, Cologne, Germany). The CAG-LSL-ALK(F1174L)-IRES-Fluc vector [LSL-ALK(F1174L)] was introduced into the ROSA26 locus of C57BL/6 embryonic stem cells by recombinase-mediated cassette exchange. Recombinant clones were isolated, validated by Southern blotting and chimera were generated by injection into blastocysts. Chimera were crossbred to C57BL/6NTac and offspring positive for the ALK(F1174L) was obtained and maintained by backcrossing on C57BL/6NTac mice. Further details about generation and characterization of the C57BL/6NTac-Gt(ROSA)26Sortm3196(CAG-ALK^*^F1174L)Arte mouse strain will be published elsewhere (Schulte et al., unpublished).

### Animal Breeding and Genotyping

The *LSL-ALK-F1174L* transgenic mice were crossed with *Sox10-Cre* mice (54). The mouse pairs were let to stand one night and the midday of the following day was considered as E0.5. Pregnant mice were killed by cervical dislocation for embryos collection. Embryos were fixed in Histofix (Roti® Histofix 4% formaldehyde, Roth) from 15 min at room temperature (RT) to overnight at +4°C, depending on embryos size, and incubated overnight in 30% sucrose (in 1x PBS) for tissue preservation. Then, embryos were embedded in optimal cutting temperature (OCT, Sakura Finetek) compound and frozen at −80°C. This study was carried out in accordance with the guidelines of the Swiss Animal Protection Ordinance and the Animal Experimentation Ordinance of the Swiss Federal Veterinary Office (FVO). The protocol was approved by the veterinary authorities of Canton of Zurich.

Genomic DNA was extracted from the tip of embryos tails and genotyping was performed by PCR using the following primers to detect Sox10-Cre and LSL-ALK-F1174L transgenes: Cre-for: 5′-CTATCCAGCAACATTTGGGCCAGC-3′ and Cre-rev: 5′-CCAGGTTACGGATATAGTTCATGAC-3′; LSL-ALK-for: 5′-CCATCAGTGACCTGAAGGAGG-3′ and LSL-ALK-rev: 5′-CACGTGCAGAAGGTCCAGC-3′.

Cycling condition were: 5′ at 94°C, 35 cycles of 40″ at 94°C, 40′′ at 55°C (Cre) or 60°C (ALK) and 1′ at 72°C, followed by 7′ at 72°C. Embryos carrying either the Sox10-Cre or LSL-ALK-F1174L transgene, or none are referred to as *WT* embryos, while embryos carrying both transgenes are referred to as *Sox10-Cre;LSL-ALK-F1174L* embryos.

### Immunofluorescence Analyses

Transverse cryosections of 10 μm were made using a cryostat and every fifteenth or twentieth embryonic section were deposited on the same glass slide (~10 sections/slide), separating each section by 150 or 200 μm, respectively.

For immunofluorescence (IF) analyses embryos sections were fixed in 4% paraformaldehyde for 5 min, then washed in PBS (3 × 5 min, same for all following washes) and treated for permeabilization with 0.5% Triton X-100 in PBS for 5 min. After washes, slides were incubated with a blocking solution (5% Goat serum, 0.1%Triton X-100 in PBS) for 45 min at RT, and then overnight at 4°C with primary antibodies diluted in PBS. The next day, after washes, corresponding secondary antibodies (diluted at 1/500 in PBS) were added for 1 h at RT, and then incubated with fluorescent mounting medium for 30 min (Dako, 1/5000). The following primary antibodies were used to detect Phox2b (Santa Cruz Biotechnology, #sc-376997, 1/100), Sox10 (Cell Signaling Technology, #89356S, 1/200); Ki67 (Abcam, #ab 15580, 1/200), TH (Abcam, #ab 76442, 1/500), βIII-tubulin (Cell Signaling Technology, #5568, 1/200), ALK (Cell Signaling, D5F3® #3633, 1/100); and revealed using the secondary Ab: Goat-a-Mouse-Alexa Fluor® 488 (ThermoFisher scientific, #A-11001), Goat-a-Rabbit- Alexa Fluor ® 647 (ThermoFisher scientific, #A-21246), Goat-a-Chicken-Alexa Fluor® 647 (Abcam, #ab 150175).

### *In situ* Hybridization

ISH experiments were performed using the RNAscope® technology (Advanced Cell Diagnostics, Inc) at the Histology Core Facility of the EPFL. RNAscope Multiplex Fluorescent V1 assay (Advanced Cell Diagnostics, Cat. No. 320850) was performed according to manufacturer's protocol on 10 μm fixed frozen cryosections, hybridized with the probes Mm-Alk1-C1 (ACD, Cat. No. 501131), Mm-Ascl1-CDS-C3 (ADC, Cat. No. 476321-C3), Mm-Insm1-C1 (ADC, Cat. No. 430621), 3Plex positive control Mm-Ppib (ACD, Cat. No. 313911), and negative control DapB (ACD, Cat. No. 310043) at 40°C for 2 h and revealed with Atto550 for C1 and Atto647 for C3.

### Imaging and Quantitative Analyses of Sympathetic Ganglia

Images for IF and ISH analyses were acquired using a fluorescence microscope (Leica DFC 345 FX, Leica). For each embryo, one slide was analyzed for each staining conditions. SG were defined as Phox2b^+^ regions near the dorsal aorta as analyzed by IF staining. To estimate the proportion of the different cell populations, all embryo sections of the slide presenting SG were analyzed. Cells were counted in SG bilaterally. For IF analyses, three *WT* embryos were analyzed for both E10.5 and E11.5, while three and two *Sox10-Cre;LSL-ALK-F1174L* embryos were analyzed for E10.5 and E11.5, respectively for Sox10/Phox2b, Phox2b/TH, Phox2b/βIII-tubulin, and Phox2b/Ki67 double stainings. The precise numbers of SG sections analyzed for each staining are indicated in the respective Figure Legends.

Areas of SG sections were quantified using Image J software (National Institute of Health, USA). The SG surfaces delimited by Phox2b positive regions was measured using the Sox10/Phox2b co-staining analyses and are given in arbitrary unit (AU). IF labeled cell populations were manually counted using the Leica Microsystem LAS AF software (Version 2.6.0, build 7266). Cell proportions were calculated by counting the number of cells comprised in of each cell population normalized to the total number of cells investigated per each SG sections. These later correspond to the total number of cells/SG section for Sox10/Phox2b staining or total number of Phox2b+ cells/SG section for βIII-tubulin/Phox2b, TH/Phox2b, and Ki67/Phox2b staining. All SG sections analyzed were independently represented on the graphs using Box-plot representation, illustrating the minimum to maximum and the median value of measurements. Quantifications of TH/h-ALK co-staining were performed using a confocal microscope (Zeiss LSM 710) and the Zeiss Blue software.

### Statistical Analysis

Statistical analyses were performed using GraphPadPrism 6.0 (GraphPad Software Inc., San Diego, CA, USA). D'Agostino-Pearson normality test was performed for each data set, and depending on data distribution, they were analyzed with unpaired two-tailed parametric *t*-test or non-parametric Mann Whitney test to compare two different conditions, or by one-way Anova or Kruskal-Wallis test for multiple comparisons. Only statistically significant comparisons are indicated in the graphs.

## Ethics Statement

This study was carried out in accordance with the guidelines of the Swiss Animal Protection Ordinance and the Animal Experimentation Ordinance of the Swiss Federal Veterinary Office (FVO). The protocol was approved by the veterinary authorities of Canton of Zurich.

## Author Contributions

LV performed all major experimental work. LV and MG performed *in vivo* experiments. LV and AM-M analyzed the data. JS produced the *LSL-ALK-F1174L* mouse model. AM-M and OS designed and coordinated experiments. LV and AM-M prepared figures and drafted the manuscript. LV, RR, and AM-M interpreted the data and edited the manuscript. All authors read, commented and approved the final manuscript.

### Conflict of Interest Statement

The authors declare that the research was conducted in the absence of any commercial or financial relationships that could be construed as a potential conflict of interest.
