# Supplementary material for: Expression of the Neuroblastoma-Associated ALK-F1174L Activating Mutation During Embryogenesis Impairs the Differentiation of Neural Crest Progenitors in Sympathetic Ganglia
Source: Front Oncol. 2019 Apr 16;9:275. doi: 10.3389/fonc.2019.00275 (PMC6477091; doi:10.3389/fonc.2019.00275)
Supplement: Supplementary file 1 [file Data_Sheet_1.docx]

Supplementary Material

**Expression of the neuroblastoma-associated ALK-F1174L activating mutation during embryogenesis impairs the differentiation of neural crest progenitors in sympathetic ganglia**

Vivancos Stalin Lucie, Gualandi Marco, Schulte Johannes Hubertus, Renella Raffaele, Shakhova Olga, Mühlethaler-Mottet Annick^*^

*** Correspondence:** Mühlethaler-Mottet Annick: [Annick.Muhlethaler@chuv.ch](mailto:Annick.Muhlethaler@chuv.ch)

## Supplementary Figures

**DAPI**

**h-ALK**

**DAPI**

**h-ALK**

Neural

tube

Neural

tube

**Supplementary Figure 1.** **Expression pattern of the ALK-F1174L transgene (h**-**ALK) in *Sox10-Cre;LSL-ALK-F1174L* embryos.** Representative images of IF staining for h-ALK (yellow) at E10.5 (x10 magnification) in two *Sox10-Cre;LSL-ALK-F1174L* embryos.

**Phox2b**

**Insm1**

**Ascl1**

**Supplementary Figure 2. Analysis of Ascl1 and Insm1 expression in SG at E10.5.** Representative images of ISH staining for Ascl1 (purple) and Insm1 (yellow) in *WT* and *Sox10-Cre;LSL-ALK-F1174L* embryos at E10.5 (x40  magnification). Phox2b IF (green) staining was applied after ISH to highlight SG. SG are surrounded by white circle. Numbers of embryos analyzed: *WT* n=2 and *Sox10-Cre;LSL-ALK-F1174L* n=3.
